# Distinct Expression Pattern and Post-Transcriptional Regulation of Cell Cycle Genes in the Glandular Epithelia of Avian Ovarian Carcinomas

**DOI:** 10.1371/journal.pone.0051592

**Published:** 2012-12-07

**Authors:** Jin-Young Lee, Wooyoung Jeong, Ji-Hye Kim, Jinyoung Kim, Fuller W. Bazer, Jae Yong Han, Gwonhwa Song

**Affiliations:** 1 WCU Biomodulation Major, Department of Agricultural Biotechnology, Seoul National University, Gwanak-gu, Seoul, Korea; 2 Center for Animal Biotechnology and Genomics and Department of Animal Science, Texas A&M University, College Station, Texas, United States of America; Western University, Canada

## Abstract

The cell cycle system is controlled in a timely manner by three groups of cyclins, cyclin dependent kinases and cyclin dependent kinase inhibitors. Abnormal alterations of cell cycle regulatory mechanisms are a common feature of many diseases including numerous tumor types such as ovarian cancer. Although a variety of cell cycle regulatory genes are well known in mammalian species including human and mice, they are not well studied in avian species, especially in laying hens which are recognized as an excellent animal model for research relevant to human ovarian carcinogenesis. Therefore, in the present study, we focused on comparative expression and regulation of expression of candidate genes which might be involved in the cell cycle program in surface epithelial ovarian cancer in laying hens. Our current results indicate that expression levels of cell cycle gene transcripts are greater in cancerous as compared to normal ovaries. In particular, cyclin A2 (*CCNA2*), *CCND1*, *CCND2*, *CCND3*, *CCNE2*, cyclin dependent kinase 1 (*CDK1*), *CDK3*, *CDK5*, cyclin dependent kinases inhibitor 1A (*CDKN1A*) and *CDKN1B* were upregulated predominantly in the glandular epithelia of cancerous ovaries from laying hens. Further, several microRNAs (miRs), specifically *miR-1798*, *miR-1699*, *miR-223* and *miR-1744* were discovered to influence expression of *CCND1*, *CCNE2*, *CDK1*, and *CDK3* mRNAs, respectively, via their 3′-UTR which suggests that post-transcriptional regulation of gene expression influences their expression in laying hens. Moreover, *miR-1626* influenced CDKN1A expression and *miR-222, miR-1787* and *miR-1812* regulated CDKN1B expression via their 3′-UTR regions. Collectively, results of the present study demonstrate increased expression of cell cycle-related genes in cancerous ovaries of laying hens and indicate that expression of these genes is post-transcriptionally regulated by specific microRNAs.

## Introduction

Epithelial ovarian cancer (EOC), the most lethal gynecological malignancy, claims the lives of over 15,000 women and 22,000 are diagnosed with the disease in the US each year [1]. However, over 75% of woman diagnosed are at an advanced stage of EOC, because it is generally asymptomatic and there is no specific biomarker(s) for early detection [2]. Therefore, to prevent and cure this lethal disease and to improve the long-term survival of patients with EOC, the most promising approach is to identify markers for early diagnosis. To overcome the problem that EOC is rarely detected at an early stage, many animal models have been developed, but they have not proven to be successful. For instance, genetically manipulated rodent models have been used to elucidate some aspects of the pathogenesis and etiologies of EOC; however, the non-spontaneous nature of their ovarian cancer limits their clinical relevance [3], [4], [5]. In fact, the laying hen is the only animal that spontaneously develops ovarian cancer of the surface epithelium of the ovaries at a high rate, as also occurs in women [6]. Thus, the laying hen is a unique animal model for human EOC research aimed at development of a biomarker(s) for detection and early diagnosis, as well as for discovery of anti-cancer drugs/biomaterials for prevention and treatment of this deadly disease.

The cell cycle in most eukaryotic cells includes a series of coordinated events consisting of cell growth, replication of genetic material, segregation of the duplicated chromosomes and cell division [7]. In general, the cell division cycle in mammals is precisely and harmoniously regulated in a timely manner by different active heterodimeric complexes that include cyclin dependent kinases (CDKs) and their cognate cyclin partners, as well as CDK inhibitors (CDKIs) [8]. Thus, tumor development frequently results when there is deregulation of the cell cycle control system including abnormal regulation of expression of cell cycle genes [1]. In human cancerous tissues, such as neoplasms, different families of cell cycle genes and regulators are frequently mutated and dysfunctional [8]. Although expression and functional roles of many CDKs, cyclins and CDKIs are well studied in mammalian species, including humans and mice, little is known about their expression and regulation in avian species, especially laying hens.

MicroRNAs (miRNAs) are endogenous non-coding short RNAs involved in various biological processes that regulate gene expression via degradation or inhibition of expression of target mRNAs. The involvement of miRNA-mediated regulatory mechanisms affecting gene transcription and translation in human cell cycle progression has been reported [9], [10], [11]. Indeed, miRNA-based fine tuning of expression of cell cycle genes is very important because improper cell cycle control is likely to lead to initiation and development of proliferative diseases, such as cancer. Although numerous miRNAs have been indentified in chickens, the functional aspects of most chicken miRNAs are not known and reports of miRNA-mediated post-transcriptional mechanism regulating cell cycle progression in chickens are not available. Understanding the target spectrum of cell cycle-related miRNAs and their functional interactions is expected to help elucidate the molecular and epigenetic regulatory mechanisms affecting transcriptional and translational events critical to control of the cell cycle and progression into carcinogenesis. Therefore, the objectives of this study with laying hens were to determine: 1) the expression of cyclins, CDKs and CDKIs in normal and cancerous ovaries; and 2) whether cyclins, CDKs and CDKIs are regulated by post-transcriptional actions of specific microRNAs (miRs) using a miR target validation assay. Our results confirm that the laying hen is a unique model for the research on human ovarian cancer and cell cycle-related genes and that regulatory factors for cell cycle-related genes play a key role in ovarian carcinomas. These cell cycle-related genes may be important targets for discovery of a biomarker(s) for diagnosis and evaluation of therapeutics designed to treat EOC in women.

## Results

### Comparative Expression of Cyclin Genes in Normal and Cancerous Ovaries from Laying Hens

**Figure 1:**
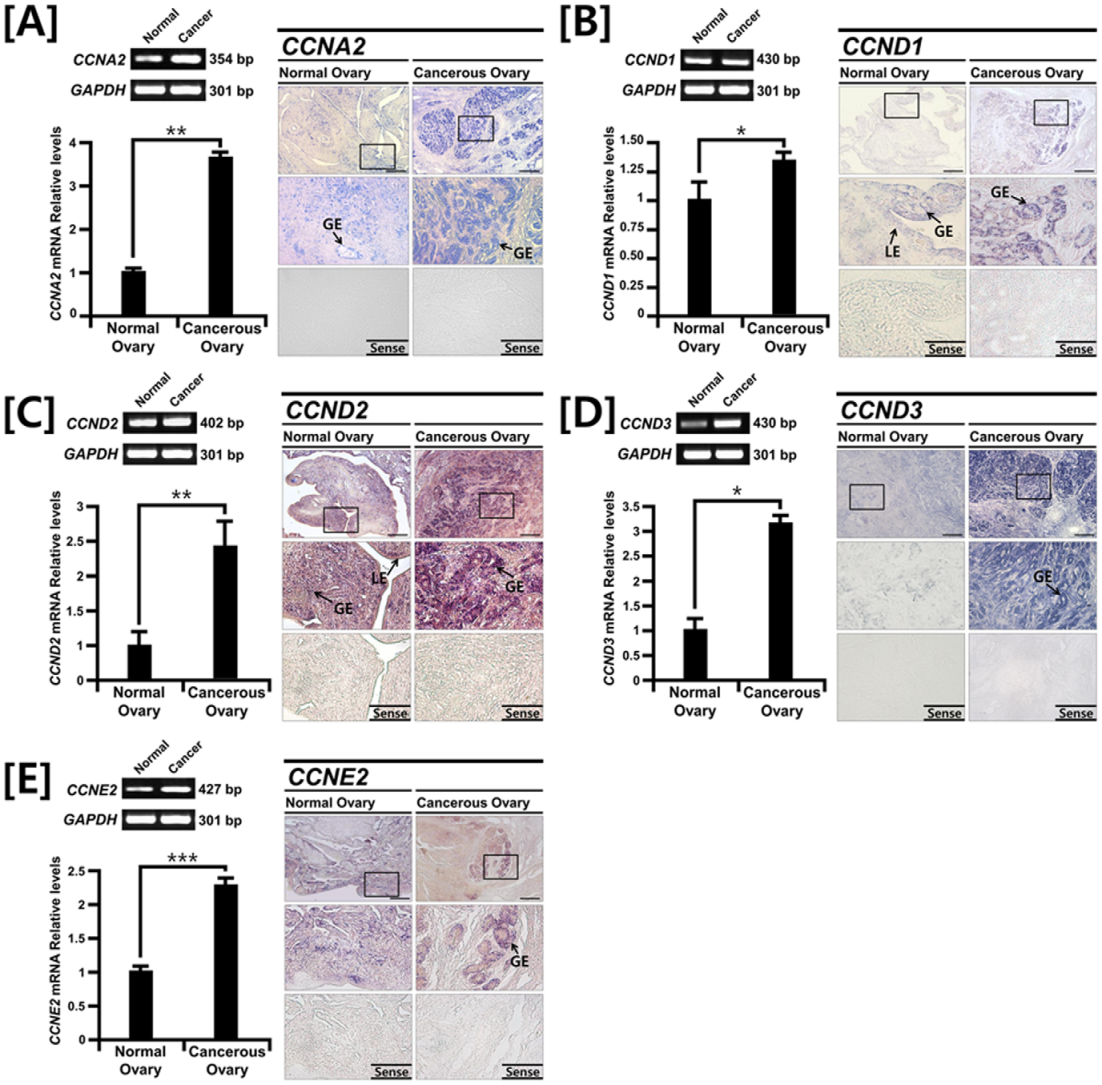
Comparative expression patterns for *CCNA2, CCND1, CCND2, CCND3* and *CCNE2* mRNAs in normal and cancerous ovaries from laying hens. RT-PCR and quantitative RT-PCR analysis were conducted using cDNA templates from normal and cancerous ovaries of laying hens using chicken *CCNA2, CCND1, CCND2, CCND3* and *CCNE2* and chicken *GAPDH* specific primers (left panel). These experiments were conducted in triplicate and values normalized to those for GAPDH. *In situ* hybridization analysis indicates cell specific expression patterns for *CCNA2, CCND1, CCND2, CCND3* and *CCNE2* mRNAs in both normal and cancerous ovaries from laying hens (right panel). See *Materials and Methods* for complete description.

To determine tissue-specific expression patterns of the cell cycle-related genes in normal (n = 5) and cancerous (n = 10) ovaries from laying hens, we performed RT-PCR, and quantitative PCR analyses. As illustrated in Figure 1, expression of cyclin A2 (*CCNA2*), *CCND1*, *CCND2*, *CCND3* and *CCNE2* mRNAs was 3.42- (*P*<0.01), 1.32- (*P*<0.05), 2.41- (*P*<0.01), 3.31- (*P*<0.05) and 2.36-fold (*P*<0.001) greater in cancerous ovaries from hens. Next, cell-specific localization of these genes in the normal and cancerous ovaries was determined using *in situ* hybridization analysis. The mRNAs for *CCNA2, CCND1, CCND2, CCND3 and CCNE2* were localized predominantly to the glandular epithelium (GE) in cancerous ovaries, but there was very weak or no detectable expression of these genes in the luminal epithelium (LE), stromal cells or blood vessels in normal and cancerous ovaries.

### Comparative Expression of Cyclin Dependent Kinase and Cyclin Dependent Kinase Inhibitor Genes in Normal and Cancerous Ovaries of Laying Hens

**Figure 2:**
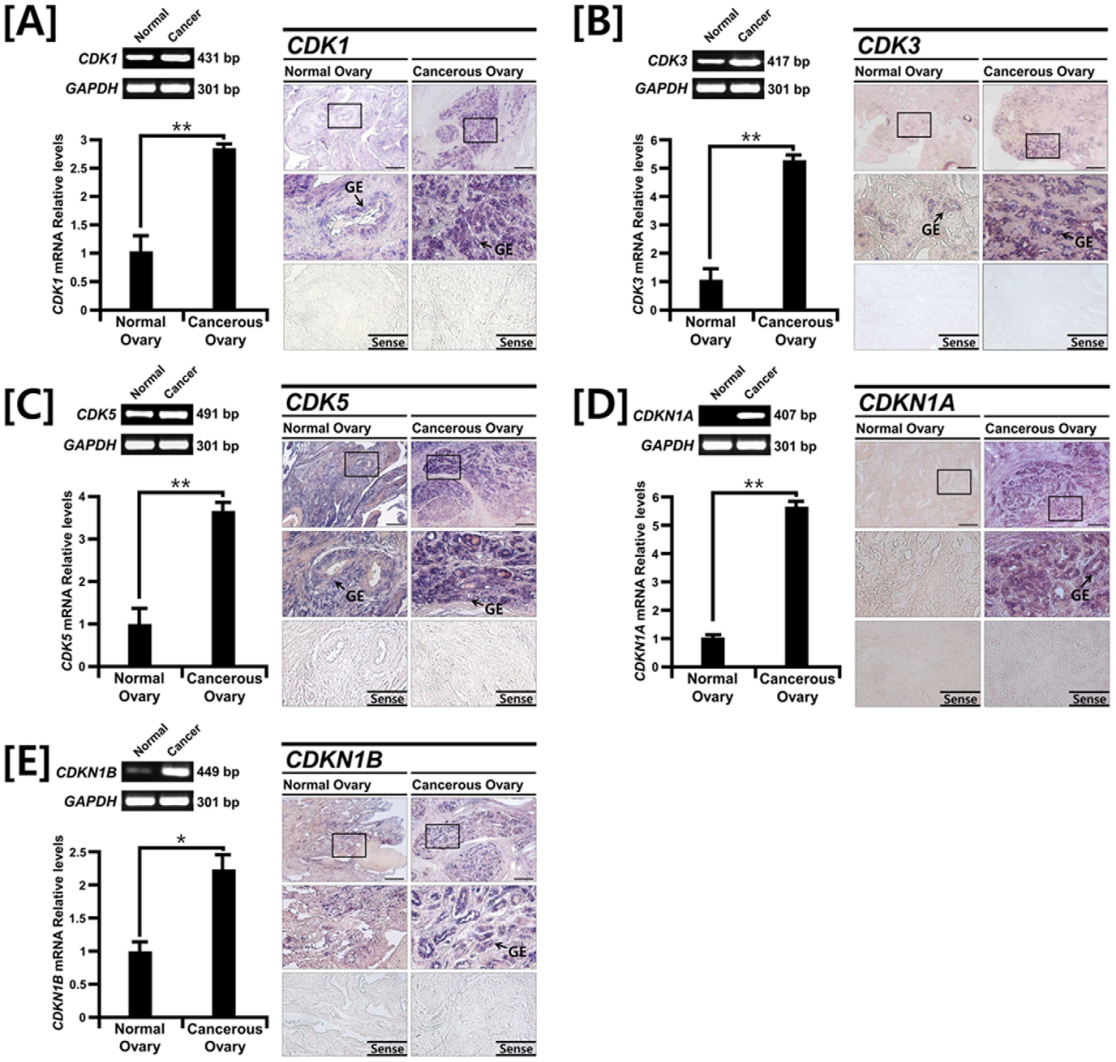
Comparative expression patterns for *CDK1, CDK3, CDK5, CDKN1A* and *CDKN1B* mRNAs in normal and cancerous ovaries from laying hens. RT-PCR and quantitative RT-PCR analyses were conducted using cDNA templates from normal and cancerous ovaries of laying hens with chicken *CDK1, CDK3, CDK5, CDKN1A* and *CDKN1B* and chicken *GAPDH* specific primers (left panel). These experiments were conducted in triplicate and normalized to values for GAPDH. *In situ* hybridization analysis indicates cell specific expression patterns of *CDK1, CDK3, CDK5, CDKN1A* and *CDKN1B* mRNAs both normal and cancerous ovaries from laying hens (right panel). See *Materials and Methods* for complete description.

As shown in Figure 2, the results from RT-PCR and quantitative PCR analyses showed that expression of mRNAs for cyclin dependent kinase 1 (*CDK1*), *CDK3* and *CDK5* were 2.87- (*P*<0.01), 5.18- (*P*<0.01) and 3.66-fold (*P*<0.01) greater in cancerous ovaries from hens, respectively. Interestingly, cyclin dependent kinase inhibitor 1A (*CDKN1A*) and *CDKN1B* mRNAs were 5.62- (*P*<0.01) and 2.31-fold (*P*<0.05) more abundant in cancerous as compared with normal ovaries. *In situ* hybridization analyses demonstrated that expression of *CDK1*, *CDK3*, *CDK5*, *CDKN1A* and *CDKN1B* mRNAs was abundant in GE and to a much lesser extent in stromal cells of cancerous ovaries, whereas there was very little or no expression of these genes in normal ovaries.

### Post-transcriptional Regulation of Specific Cell Cycle Regulatory Genes by Chicken microRNAs

**Figure 3:**
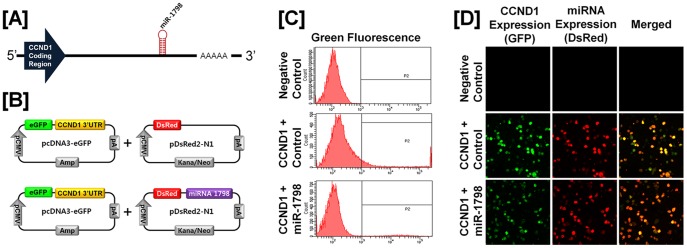
*In vitro* target assay for *microRNAs* on the *CCND1* transcript. [A] Diagram of *miR-1798* binding sites in the 3′-UTR of the *CCND1* gene. [B] Expression vector maps for eGFP within the 3′-UTR of the *CCND1* gene and Ds-Red within each miRNA. The 3′-UTR of the *CCND1* transcript was subcloned between the *eGFP* gene and the polyA tail to generate the fusion construct of the GFP transcript following the miRNA target 3′-UTR (pcDNA-eGFP-3′UTR) (upper panel) and the miRNA expression vector was designed to co-express DsRed and each miRNA (pcDNA-DsRed-miRNA) (lower panel). [C and D] After co-transfection of pcDNA-eGFP-3′UTR for the *CCND1* transcript and pcDNA-DsRed-miRNA for *miR-1798,* the fluorescence signals of GFP and DsRed were detected using FACS [C] and fluorescent microscopy [D]. See *Materials and Methods* for complete description.

**Figure 4:**
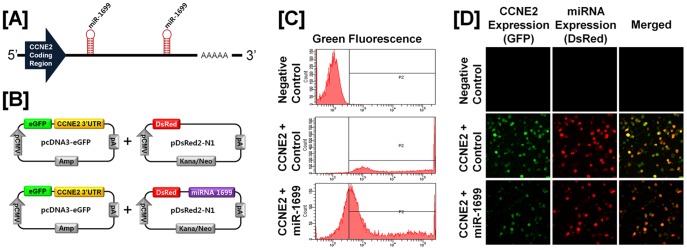
*In vitro* target assay for *microRNAs* on the *CCNE2* transcript. [A] Diagram of *miR-1699* binding sites in the 3′-UTR of the *CCNE2* gene. [B] Expression vector maps for eGFP within the 3′-UTR of the *CCNE2* gene and Ds-Red within each miRNA. The 3′-UTR of the *CCNE2* transcript was subcloned between the *eGFP* gene and the polyA tail to generate the fusion construct of the GFP transcript following the miRNA target 3′-UTR (pcDNA-eGFP-3′UTR) (upper panel) and the miRNA expression vector was designed to co-express DsRed and each miRNA (pcDNA-DsRed-miRNA) (lower panel). [C and D] After co-transfection of pcDNA-eGFP-3′UTR for the *CCNE2* transcript and pcDNA-DsRed-miRNA for the *miR-1699,* the fluorescence signals of GFP and DsRed were detected using FACS [C] and fluorescent microscopy [D]. See *Materials and Methods* for complete description.

**Figure 5:**
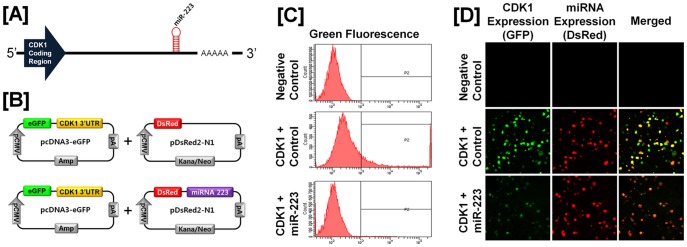
*In vitro* target assay for *microRNAs* on the *CDK1* transcript. [A] Diagram of *miR-223* binding sites in the 3′-UTR of the *CDK1*gene. [B] Expression vector maps for eGFP within the 3′-UTR of the *CDK1* gene and Ds-Red within each miRNA. The 3′-UTR of the *CDK1* transcript was subcloned between the *eGFP* gene and the polyA tail to generate the fusion construct for the GFP transcript following the miRNA target 3′-UTR (pcDNA-eGFP-3′UTR) (upper panel) and the miRNA expression vector was designed to co-express DsRed and each miRNA (pcDNA-DsRed-miRNA) (lower panel). [C and D] After co-transfection of pcDNA-eGFP-3′UTR for the *CDK1* transcript and pcDNA-DsRed-miRNA for the *miR-223,* the fluorescence signals of GFP and DsRed were detected using FACS [C] and fluorescent microscopy [D]. See *Materials and Methods* for complete description.

**Figure 6:**
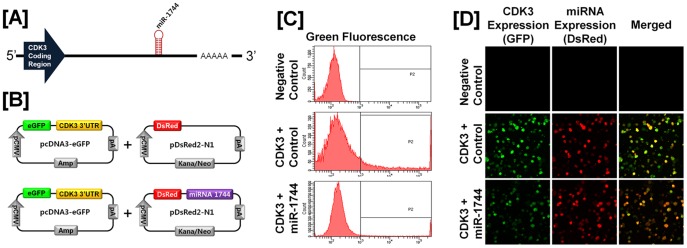
*In vitro* target assay for *microRNAs* on the *CDK3* transcript. [A] Diagram of *miR-1744* binding sites in the 3′-UTR of the *CDK3*gene. [B] Expression vector maps for eGFP within the 3′-UTR of the *CDK3* gene and Ds-Red within each miRNA. The 3′-UTR of the *CDK3* transcript was subcloned between the *eGFP* gene and the polyA tail to generate the fusion construct for the GFP transcript following the miRNA target 3′-UTR (pcDNA-eGFP-3′UTR) (upper panel) and the miRNA expression vector was designed to co-express DsRed and each miRNA (pcDNA-DsRed-miRNA) (lower panel). [C and D] After co-transfection of pcDNA-eGFP-3′UTR for the *CDK3* transcript and pcDNA-DsRed-miRNA for the *miR-1744,* the fluorescence signals of GFP and DsRed were detected using FACS [C] and fluorescent microscopy [D]. See *Materials and Methods* for complete description.

**Figure 7:**
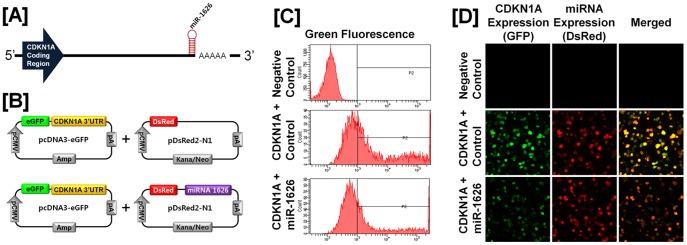
*In vitro* target assay for *microRNAs in* the *CDKN1A* transcript. [A] Diagram of *miR-1626* binding sites in 3′-UTR of the *CDKN1A* gene. [B] Expression vector maps for eGFP within the 3′-UTR of the *CDKN1A* gene and Ds-Red within each miRNA. The 3′-UTR of the *CDKN1A* transcript was subcloned between the *eGFP* gene and the polyA tail to generate the fusion construct of the GFP transcript following the miRNA target 3′-UTR (pcDNA-eGFP-3′UTR) (upper panel) and the miRNA expression vector was designed to co-express DsRed and each miRNA (pcDNA-DsRed-miRNA) (lower panel). [C and D] After co-transfection of pcDNA-eGFP-3′UTR for the *CDKN1A* transcript and pcDNA-DsRed-miRNA for the *miR-1626,* the fluorescence signals of GFP and DsRed were detected using FACS [C] and fluorescent microscopy [D]. See *Materials and Methods* for complete description.

**Figure 8:**
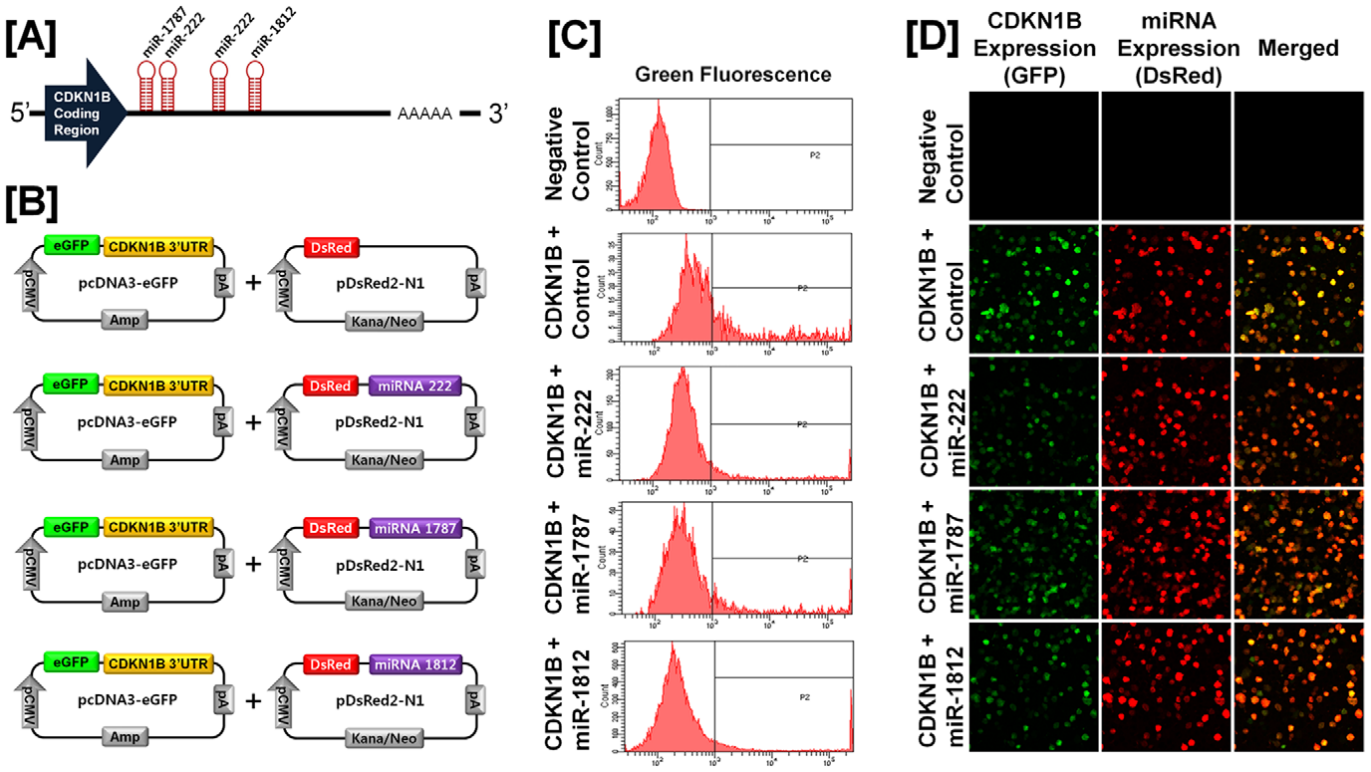
*In vitro* target assay for *microRNAs in* the *CDKN1B* transcript. [A] Diagram of *miR-222, miR-1787* and *miR-1812* binding sites in the 3′-UTR of the *CDKN1B*gene. [B] Expression vector maps for eGFP within the 3′-UTR of the *CDKN1B* gene and Ds-Red within each miRNA. The 3′-UTR of the *CDKN1B* transcript was subcloned between the *eGFP* gene and the polyA tail to generate the fusion construct of the GFP transcript following the miRNA target 3′-UTR (pcDNA-eGFP-3′UTR) (upper panel) and the miRNA expression vector was designed to co-express DsRed and each miRNA (pcDNA-DsRed-miRNA) (lower panel). [C and D] After co-transfection of pcDNA-eGFP-3′UTR for the *CDKN1B* transcript and pcDNA-DsRed-miRNA for the *miR-222, miR-1787* and *miR-1812,* the fluorescence signals of GFP and DsRed were detected using FACS [C] and fluorescent microscopy [D]. See *Materials and Methods* for complete description.

A microRNA (miR) target validation assay was used to test the hypothesis that expression of cell cycle genes is regulated at the post-transcriptional level by specific miRNAs. Analysis of potential miRNA binding sites within the 3′-UTR of the six cell cycle regulatory genes was performed using the miRNA target prediction database (miRDB; http://mirdb.org/miRDB/). This analysis revealed putative binding sites for several chicken miRNAs (*miR-1798* for *CCND1*; *miR-1699* for *CCNE2*; *miR-223* for *CDK1*; *miR-1744 for CDK3*; *miR-1626* for *CDKN1A*; and *miR-222, miR-1787* and *miR-1812* for *CDKN1B*), but not for the other four genes of interest. Therefore, we determined whether these miRNAs influenced expressions of cell cycle regulatory genes via the 3′-UTR. As illustrated in Figures 3 and 4, in the presence of *miR-1798* and miR-*1699*, the intensity and percentage of GFP-CCND1-expressing cells (12.7% in control vs. 4.2% in *miR-1798*) and GFP-CCNE2-expressing cells (96.4% in control vs. 71.4% in miR-1699) decreased (*P*<0.01). In addition, as shown in Figures 5 and 6, in the presence of *miR-223* and miR-*1744*, the intensity and percentage of GFP-CDK1-expressing cells (17.2% in control vs. 1.3% in miR-223) and GFP-CDK3-expressing cells (16.1% in control vs. 6.8% in *miR-1744*) were decreased (*P*<0.01). Moreover, in the presence of *miR-1626*, the intensity and percentage of GFP-CDKN1A-expressing cells (54.6% in control vs. 34.7% in *miR-1626*) were decreased (*P*<0.01) (Figure 7). In addition, for CDKN1B, in the presence of *miR-222, miR-1787* and *miR-1812,* the intensity and percentage of GFP-CDKN1B-expressing cells (29.0% in control vs. 15.6% in *miR-1787,* 12.6% in *miR-1812,* 9.8% in *miR-222*) were decreased (*P*<0.01) (Figure 8). These results indicate that at least one to three miRNAs bind directly to the cell cycle-related gene transcripts to regulate expression.

## Discussion

Results of the present study provide the first evidence of significant differences in expression of *CCNA2, CCND1, CCND2, CCND3, CCNE2, CDK1, CDK 3, CDK5, CDKN1A* and *CDKN1B* genes in cancerous as compared to normal ovaries of laying hens. In addition, our results indicate that several microRNAs (miRs), specifically *miR-222, miR-223, miR-1626, miR-1699*, *miR-1744, miR-1787, miR-1798* and *miR-1812* interact with sites in the 3′-UTR of the cell cycle genes and regulatory factors affecting cell cycle genes including *CCND1, CCNE2, CDK1, CDK3, CDKN1A* and *CDKN1B* to influence post-transcriptional regulation of its expression in laying hens. These results support our hypothesis that cell cycle genes are critical regulators for growth and developmental aspects of epithelial cells of the ovaries of hens and that there is dysregulation of their level of expression as ovaries of laying hens transition from a normal to a cancerous state.

In the United States, ovarian cancer is the most common malignancy in the female genital tract and the fifth leading cause of cancer-related deaths among women. The surface epithelial-derived ovarian cancer (EOC) accounts for 90% of all ovarian cancers [12]. The idea that the repeated rupture of the ovarian surface epithelium during the monthly ovulation event in women may contribute to or accelerate the incidence of the EOC was proposed by Fathalla about four decades ago [13]; however, the etiology and pathology of the EOC is complicated and not fully understood. Results of a number of epidemiological and histological studies strongly support the idea that there is an increased incidence of EOC dependent on the frequency of ovulation and other factors associated with the reproductive tract [14]. However, there is evidence suggesting that serous-type carcinomas do not begin as precursor lesions in the fimbria of the fallopian tube in women and then spread to the surface epithelium of the ovary or shed into the peritoneal cavity [15], [16], [17]. In addition, there is a report indicating that EOC is caused from instability of the copy number of a certain gene, but not from the mutation of the gene(s) [18]. At present, the laying hen is an established animal model for study of EOC to elucidate the pathogenesis and etiologies of EOC because they spontaneously develop surface epithelium-derived ovarian cancer at a high rate as occurs in women [6]. There are no reports that ovarian carcinoma has its origin in the oviduct (fallopian tube) of laying hens. Further, except for serous carcinoma, there are no reports of the other three types of ovarian cancer including endometrioid, mucinous and clear cell carcinomas in humans. Therefore, future studies will investigate the origins of the different types of ovarian carcinoma are regulatory mechanism(s) that govern the initiation and development of ovarian carcinogenesis in women and laying hen.

Cyclins are a family of proteins that control the cell cycle by binding and activating cyclin-dependent kinases. As illustrated in Figures 1, D-type cyclins, which are G_1_ phase regulators of the cell cycle [1], [8], such as CCND1, CCND2 and CCND3 are predominantly found in cancerous ovaries, but there was weak or little expression in normal ovaries of laying hens in the present study. In humans, CCND1 is frequently overexpressed in a variety of tumor types and is associated with carcinogenesis and metastasis [19]. Dhar and colleagues reported that expression of CCND1 was up-regulated in about 90% of patients with EOC and expressed mainly in both borderline and invasive tumours without any association between immunoreactive protein overexpression and stage of tumor differentiation or grade of tumor [20]. In the present study, we found *CCND1, CCND2 and CCND3* mRNAs in the nucleus and the cytoplasm of epithelial cells of normal ovaries, but exclusively in the cytoplamic compartment of the epithelial cells in cancerous ovaries of laying hens. This result is consistent with deregulation of CCND1 expression leading to localization of the protein in both the cytoplasmic and nuclear compartments of cells from cancerous ovaries [20]. Interestingly, although CCND2 and CCND3 are not overexpressed in human ovarian cancer [21], messenger RNA expression levels for both CCND2 and CCND3 were significantly upregulated in cancerous ovaries of laying hens. The difference in expression of these genes between humans and laying hens should be elucidated. Expression of *CCNE2* mRNA, a G_1_-S phase regulator [7], in cancerous ovaries from hens was 2.36-fold (*P*<0.001) greater than in normal ovaries, and mainly detected in the glandular epithelium (GE). This result supports the idea that CCNE protein is valuable prognostic factors for EOC patients because amplification and over-expression of the *CCNE1* gene occurs in many cases with a gradual increase from benign to borderline to malignant tumors [21], [22]. In addition, *CCNA2* mRNA, an S phase regulators [1], [7], was found predominantly in GE and its expression was 3.42-fold (*P*<0.01) greater in cancerous ovaries of laying hens. In humans, CCNA expression increased in the ovarian carcinoma cell line compared with normal cells [23] and CCNA protein was detected mainly in serous and endometrioid carcinomas, but not in mucinous and clear cell carcinomas [24].

Cyclin dependent kinases (CDKs) are the catalytic subunits of a large family of heterodimeric serine/threonine protein kinases that have essential roles in controlling progression of the cell cycle [25]. In the present study, we found that *CDK1, CDK3* and *CDK5* mRNAs were up-regulated predominantly in GE of cancerous ovaries, but there was weak or no expression of these mRNAs in normal ovaries of laying hens. In general, CDK1 has an essential role in the transition of cells into mitosis under normal circumstances as a component of the cell cycle control system and it is also involved in rapid arrest in G_2_ phase in response to DNA damage [26]. Overexpression of CDK1 is detected in 79% of EOC patients, but not in benign epithelial tumors or normal epithelial tissues in women [27]. In addition, overexpression of CDK2 was found in only 6% of EOC patients, but its level of expression was positively correlated with CCNE abundance, suggesting that overexpression of both CDK2 and CCNE is significantly associated with development of malignant ovarian tumors [22], [28]. Furthermore, CDK4 is overexpressed in 14% to 15% of ovarian tumors [29] and its activity in malignant ovarian tumors is significantly greater than in benign tumors [22]. These results suggest that CDK4 activity play important roles in ovarian carcinogenesis. Collectively, results of the present study strongly indicate that CDK activity is regulated by cyclin synthesis and degradation, and that orderly progression through the cell cycle requires coordinated activation of the CDK proteins by binding to the cyclin partner [8]. Furthermore, the results confirm that the laying hen is an appropriate animal model for identifying and developing biomarkers for early diagnosis and evaluation of therapeutics for treatment of ovarian cancer [5], [6], [30], [31], [32], [33], [34], [35], [36].

MicroRNAs, short and noncoding RNAs of 18 to 23 nucleotides in length, regulate complex patterns of gene expression post-transcriptionally and are capable of defining and altering cell fate by silencing translational of gene transcripts through cleavage of their target mRNAs through base pairing at partially or fully complementary sites [37]. As shown in Figures 3, 4, 5, 6, *miR-1798* and *miR-1699* influence the expression of CCND1 and CCNE2, respectively, while *miR-223* and *miR-1744* regulate expression of CDK1 and CDK3, respectively. By regulating post-transcriptional events, miRs affect function of a number of cellular processes in development, differentiation and oncogenesis [38], [39], [40]. Results of the present study demonstrated that *miR-1798* inhibits expression of CCND1 in laying hens. In human ovarian cancer, deregulation of CCND1 expression mainly occurs without any gene amplification [20]. Thus, we suggest dysfunction of *miR-1787* leads to the overexpression of CCND1 in cancerous ovaries of laying hens. In fact, deregulation of miRs is generally considered to be a prerequisite for initiation and progression of carcinogenesis in humans. For instance, functional overexpression of *miR-31* inhibits proliferation and induces apoptosis in a variety of serous-type ovarian cancer cell lines, such as SKOV3, with a dysfunctional p53 pathway [41]. In addition, miR expression of transcriptional targets of p53 (i.e. *miR-34b* and *miR-34c*) is markedly down-regulated in human EOC tissues [42]. These results indicated that miRs may be useful in predicting outcomes of many diverse carcinomas, including EOC.

As shown in Figure 2, there is overexpression of cyclin dependent kinase inhibitor 1A (*CDKN1A*) and *CDKN1B* mRNAs in cancerous ovaries of laying hens. CDKN1A and CDKN1B (also known as p21/WAF1 and p27, respectively) are potent CDK inhibitors and act as regulators in the G_1_ phase of the cell cycle. In humans, aberrant expression of CDKN1A and/or CDKN1B in various carcinomas of lung, colorectum, cervix, head and neck leads to carcinogenesis by blocking DNA synthesis and inhibiting cell growth [43], [44], [45], [46]. In other words, loss of both genes may contribute to tumor progression. For instance, loss of CDKN1B protein was significantly associated with a relatively shorter time to cell cycle progression and decreased overall survival rates in patients with advanced ovarian cancer [47]. These results indicate that both CDKN1A and CDKN1B are potential prognostic markers to predict progression of EOC and survival in EOC patients. However, it has been reported that CDKN1B has dual functional roles during tumorigenesis. In mice, Cdkn1b acts as a tumor suppressor due to its cyclin-CDK regulatory function and it acts as an oncogene through a cyclin-CDK-independent function [48]. These results may explain why *CDKN1A* and *CDKN1B* genes have controversial patterns of expression pattern in human and mouse tumors. Therefore, we suggest that synchronous up-regulation of *CDKN1A* and *CDKN1B* genes with other *CDK* genes in the present study may be caused by a CDK-independent-oncogenic-function of both CDKN1A and CDKN1B instead of an inhibitory function of these CDKs [48]. Future research is required to gain a better understanding of the CDK-independent-oncogenic-function of both genes in ovarian carcinogenesis in laying hens. In addition, our miR target validation assay demonstrated that *miR-1626* regulates *CDKN1A* expression and *miR-222, miR-1787* and *miR-1812* influence post-transcriptional modification of transcripts of the *CDKN1B* gene. These results suggest that down-regulation of these miRs might contribute to the overexpression of cell cycle genes and regulatory factors in chicken ovarian cancer and to transcriptional deregulation of many genes in the genome, that may lead to uncontrolled carcinogenesis.

Collectively, results of the present study indicate that overexpression of cell cycle-related genes (i.e. cyclins, their associated kinases and inhibitors) may be involved in uncontrolled cell proliferation, growth and loss of function in cells that leads to ovarian tumorigenesis in laying hens. Furthermore, post-transcriptional regulation of the specific miRs that influence expression of cell cycle genes likely leads to an alternative mechanism(s) for regulation of their expression. Although results of this study indicate that various miRs might be involved in many different oncogenic/carcinogenic pathways, details of altered expression patterns and their relevance to EOC remain to be elucidated. Thus, further research is clearly required to unravel the mechanism(s) for post-transcriptional regulation of cell cycle-dependent gene expression and different oncogenic pathways leading to ovarian carcinogenesis in women and in laying hens.

## Materials and Methods

### Experimental Animals and Animal Care

The experimental use of chickens for this study was approved by the Institute of Laboratory Animal Resources, Seoul National University (SNU-070823-5). White Leghorn (WL) laying hens were subjected to standard management practices at the Seoul National University Animal Farm, Seoul, Republic of Korea. All chickens were exposed to a light regimen of 15 h light and 9 h dark with *ad libitum* access to feed and water, as well as standard management practices for feeding and husbandry.

### Tissue Samples

In this study, a total of 136 laying hens (88 over 36 months of age and 48 over 24 months of age), which had completely stopped laying eggs, were euthanized for biopsy and cancerous (n = 10) ovaries were collected. As a control, normal (n = 5) ovaries were also collected from laying hens. We examined tumor stage and the degree of metastasis in 10 hens with cancerous ovaries according to characteristic features of chicken epithelial ovarian cancers [5] (see the Table 1 of the reference). In three hens, ovarian tumor cells were classified as Stage III as they had metastasized to the gastrointestinal tract and superficial surface of the liver with profuse ascites in the abdominal cavity. In five hens, the tumors had metastasized to distant organs such as liver parenchyma, lung, gastrointestinal tract and oviduct with profuse ascites, so these were classified at Stage IV tumors. The other two hens did not have tumors in any other organs; therefore, their ovarian tumors were classified as Stage I. The collected cancerous and normal ovaries containing follicles, glands, stromal cells and blood vessels were frozen in liquid nitrogen or fixed in 4% paraformaldehyde for further analyses. Frozen tissue samples were cut into 5- to 7-mm pieces before being frozen in liquid nitrogen. The other samples were cut into 10 mm pieces and fixed in 4% paraformaldehyde in PBS (pH 7.4). After 24 h, fixed tissues were changed to 70% ethanol for 24 h and then dehydrated and embedded in Paraplast-Plus (Leica Microsystems, Wetzlar, Germany). Paraffin-embedded tissues were sectioned at 5 µm and stained with hematoxylin and eosin. Epithelial ovarian cancers in laying hens were classified based on their cellular subtypes and patterns of cellular differentiation with reference to ovarian malignant tumor types in humans.

**Table 1:** Information on primers for RT-PCR analyses.

*Gene*	Sequence (5'→3'):	GenBank	Product Size (bp)
	forward and reverse	accession no.	
*CCNA2*	ATGTCAGCGATATCCACACG GCTCCATCCTCAGAACTTGC	NM_205244.1	354
*CCND1*	AGACCATCCGACGAGCCTAC GCAGCCAGATTCCATTTCAG	NM_205381.1	430
*CCND2*	AGTTGCTGTGCTGCGAGGT GCTCTTGGGGTTTGATGGAA	NM_204213.1	402
*CCND3*	CGTCTCCTACTTCCAATGCG GGTCTGTGCGTGCTTCTTCA	NM_001008453.1	430
*CCNE2*	ACCTCACTCTTCATTGCCTCC TCACAAACGGAACCATCCAC	NM_001030945.1	427
*CDK1*	GGCAGATTTTGGATTGGCTC	NM_205314.1	431
	CGAAGTATGGGTGGTTCAAGG		
*CDK3*	ACCCCAACATCGTCAAACTG GCGTCACCATCTCAGCAAAA	NM_001081706.2	417
*CDK5*	TCTGGTCTTTGAGTTCTGCGA TGGGGTAGGGCTTGTAGTCA	NM_001135786.1	491
*CDKN1A*	CGTGCAGGAACCTCTTCG TCACAGCTTGGGCTTATCG	NM_204396.1	407
*CDKN1B*	CGCAAGGAAATGGAAGAGG GTTTGATGTCGTCTCGGGC	NM_204256.2	449
*GAPDH*	TGCCAACCCCCAATGTCTCTGT TCCTTGGATGCCATGTGGACCA	NM_204305.1	301

### RNA Isolation

Total cellular RNA was isolated from frozen tissues using Trizol reagent (Invitrogen, Carlsbad, CA) according to the manufacturer’s recommendations. The quantity and quality of total RNA was determined by spectrometry and denaturing agarose gel electrophoresis, respectively.

### Semiquantitative RT-PCR Analysis

The expression of mRNAs for *cell cycle genes* in normal and cancerous ovaries of laying hens was assessed using semi-quantitative RT-PCR as described previously [49]. Information on the primer sets is provided in Table 1. The cDNA was synthesized from total cellular RNA (2 ug) using random hexamer (Invitrogen, Carlsbad, CA) and oligo (dT) primers and AccuPower® RT PreMix (Bioneer, Daejeon, Korea). The cDNA was diluted (1∶10) in sterile water before use in PCR. The primers, PCR amplification and verification of their sequences were conducted as described previously [49]. After PCR, equal amounts of reaction product were analyzed using a 1% agarose gel, and PCR products were visualized using ethidium bromide staining. The amount of DNA present was quantified by measuring the intensity of light emitted from correctly sized bands under ultraviolet light using a Gel Doc™ XR+ system with Image Lab™ software (Bio-Rad).

### Quantitative RT-PCR Analysis

Total RNA was extracted from normal and cancerous ovarian tissue using TRIzol (Invitrogen) and purified using an RNeasy Mini Kit (Qiagen). Complementary DNA was synthesized using a Superscript® III First-Strand Synthesis System (Invitrogen). Gene expression levels were measured using SYBR® Green (Biotium, Hayward, CA, USA) and a StepOnePlus™ Real-Time PCR System (Applied Biosystems, Foster City, CA, USA). The *glyceraldehydes 3-phosphate dehydrogenase (GAPDH)* gene was analyzed simultaneously as a control and used for normalization of data. Each target gene and GAPDH were analyzed in triplicate. Using the standard curve method, we determined the expression quantities of the examined genes using the standard curves and C_t_ values, and normalized them to GAPDH expression values. ROX dye (Invitrogen) was used as a negative control for the fluorescence measurements. Sequence-specific products were identified by generating a melting curve in which the C_t_ value represented the cycle number at which a fluorescent signal rose statistically above background, and relative gene expression was quantified using the 2^–ΔΔ^C_t_ method [50]. For the control, the relative quantification of gene expression was normalized to the C_t_ of the control ovaries. Information on the primer sets is provided in Table 2.

**Table 2:** Information on primers for quantitative PCR analyses.

*Gene*	Sequence (5'→3'):	GenBank	Product Size (bp)
	forward and reverse	accession no.	
*CCNA2*	TATTCTGGTGGACTGGCTGG CGAACTCTGCTACTTCAGGGG	NM_205244.1	200
*CCND1*	GACTTTTGTGCGTCTGTGCG TCTTGGCAGGCTCGTAAACT	NM_205381.1	198
*CCND2*	CAAGCACAGATGTGGACTGC CTGGTCCAGTTCCTCAATGG	NM_204213.1	131
*CCND3*	GATGGAGCTGGTGAAGAAGC GCTTCAGGCTCTCAGCTAGG	NM_001008453.1	254
*CCNE2*	GCTGCACTCTGCCACTATACC ATTCACAAACGGAACCATCC	NM_001030945.1	105
*CDK1*	AGGTATCGTCTTCTGCCATTCA	NM_205314.1	110
	GAGCCAATCCAAAATCTGCC		
*CDK3*	CCAGAAGGTGGAGAAGATCG GCCTGACTATGTTGGGATGC	NM_001081706.2	185
*CDK5*	CGAGAAGCTGGAGAAGATCG CCAGAGTCAGCTTCTTGTCG	NM_001135786.1	224
*CDKN1A*	GTGTCGGTGGGGCTCATC GCTTGGCGTTATCGTGGAC	NM_204396.1	144
*CDKN1B*	AAGAAGCACCGCAAGGAAAT CTGCCTGAAGTAGAAGTCGGG	NM_204256.2	138
*GAPDH*	ACACAGAAGACGGTGGATGG GGCAGGTCAGGTCAACAACA	NM_204305.1	193

### *In Situ* Hybridization Analysis

For hybridization probes, PCR products were generated from cDNA with the primers used for RT-PCR analysis. The products were gel-extracted and cloned into pGEM-T vector (Promega). After verification of the sequences, plasmids containing gene sequences were amplified with T7- and SP6-specific primers (T7:5′-TGT AAT ACG ACT CAC TAT AGG G-3′; SP6:5′-CTA TTT AGG TGA CAC TAT AGA AT-3′) then digoxigenin (DIG)-labeled RNA probes were transcribed using a DIG RNA labeling kit (Roche Applied Science, Indianapolis, IN). Tissues were collected and fixed in 4% paraformaldehyde. The tissues were embedded in paraffin and sectioned at 5 µm on APES-treated (silanized) slides. The sections were then deparaffinized in xylene and rehydrated to diethylpyrocarbonate (DEPC)-treated water through a graded series of alcohol. The sections were treated with 1% Triton X-100 in PBS for 20 min and washed two times in DEPC-treated PBS. After washing in DEPC-treated PBS, the sections were digested in TE buffer (100 mMTris-HCl, 50 mM EDTA, pH 8.0) containing 5 µg/ml Proteinase K (Sigma Chemical Co., St. Louis, MO) at 37°C. After post-fixation in 4% paraformaldehyde, sections were incubated twice for 5 min each in DEPC-treated PBS and incubated in TEA buffer (0.1 M triethanolamine) containing 0.25% (v/v) acetic anhydride. The sections were incubated in a prehybridization mixture containing 50% formamide and 4X standard saline citrate (SSC) for at least 10 min at room temperature. After prehybridization, the sections were incubated with a hybridization mixture containing 40% formamide, 4X SSC, 10% dextran sulfate sodium salt, 10 mM DTT, 1 mg/ml yeast tRNA, 1 mg/ml salmon sperm DNA, 0.02% Ficoll, 0.02% polyvinylpyrrolidone, 0.2 mg/ml RNase-free bovine serum albumin and denatured DIG-labeled cRNA probe overnight at 42°C in a humidified chamber. After hybridization, sections were washed for 15 min in 2X SSC at 37°C, 15 min in 1X SSC at 37°C, 30 min in NTE buffer (10 mM Tris, 500 mM NaCl and 1 mM EDTA) at 37°C and 30 min in 0.1X SSC at 37°C. After blocking with a 2% normal sheep serum (Santa Cruz Biotechnology, Inc., Santa Cruz, CA), the sections were incubated overnight with sheep anti-DIG antibody conjugated to alkaline phosphatase (Roche, Indianapolis, IN). The signal was visualized by exposure to a solution containing 0.4 mM 5-bromo-4-chloro-3-indolylphosphate, 0.4 mM nitrobluetetrazolium, and 2 mM levamisole (Sigma Chemical Co., St. Louis, MO).

### MicroRNA Target Validation Assay

The 3′-UTR of selected genes was cloned and confirmed by sequencing. The 3′-UTR was subcloned between the eGFP gene and the bovine growth hormone (bGH) poly-A tail in pcDNA3eGFP (Clontech, Mountain View, CA) to generate the eGFP-miRNA target 3′-UTR (pcDNA-eGFP-3′UTR) fusion constructs. For the dual fluorescence reporter assay, the fusion constructs containing the *DsRed* gene and each microRNA were designed to be co-expressed under control of the CMV promoter (pcDNA-DsRed-miRNA). The pcDNA-eGFP-3′UTR and pcDNA-DsRed-miRNA (4 µg) were co-transfected into 293FT cells using the calcium phosphate method. When the DsRed-miRNA is expressed and binds to the target site of the 3′-UTR downstream of the GFP transcript, green fluorescence intensity decreases due to degradation of the GFP transcript. At 48 h post-transfection, dual fluorescence was detected by fluorescence microscopy and calculated by FACSCalibur flow cytometry (BD Biosciences). For flow cytometry, the cells were fixed in 4% paraformaldehyde and analyzed using FlowJo software (Tree Star Inc., Ashland, OR). We captured fluorescence images using a confocal laser scanning microscope and zen 2009 microscopy software (Carl zeiss, Germany) with the following settings: Lenses, 20×; Frame size, 128(X) and 128(Y); Laser settings, 3% FITC at 488 nm) and 2.8% DsRed at 555 nm; Scan time, 491 msec; Pinhole size; 1Airy unit.

### Statistical Analyses

Data for quantitative PCR were subjected to analysis of variance (ANOVA) according to the general linear model (PROC-GLM) of the SAS program (SAS Institute, Cary, NC). Data are presented as mean ± SEM unless otherwise stated. Differences in the variance between normal and each classification of cancerous ovary group were analyzed using the *F* test, and differences in the means were subjected to Student’s *t* test. Differences were considered significant at P<0.05.

## References

[1] Bovicelli A, D'Andrilli G, Giordano A (2011) New players in ovarian cancer. J Cell Physiol 226: 2500–2504. 21302305 10.1002/jcp.22662

[2] Bast RC Jr, Urban N, Shridhar V, Smith D, Zhang Z, et al (2002) Early detection of ovarian cancer: promise and reality. Cancer Treat Res 107: 61–97. 11775462 10.1007/978-1-4757-3587-1_3

[3] Vanderhyden BC, Shaw TJ, Ethier JF (2003) Animal models of ovarian cancer. Reprod Biol Endocrinol 1: 67. 14613552 10.1186/1477-7827-1-67 PMC270002

[4] Stakleff KD, Von Gruenigen VE (2003) Rodent models for ovarian cancer research. Int J Gynecol Cancer 13: 405–412. 12911715 10.1046/j.1525-1438.2003.13317.x

[5] Barua A, Bitterman P, Abramowicz JS, Dirks AL, Bahr JM, et al (2009) Histopathology of ovarian tumors in laying hens: a preclinical model of human ovarian cancer. Int J Gynecol Cancer 19: 531–539. 19509547 10.1111/IGC.0b013e3181a41613 PMC2759668

[6] Stammer K, Edassery SL, Barua A, Bitterman P, Bahr JM, et al (2008) Selenium-Binding Protein 1 expression in ovaries and ovarian tumors in the laying hen, a spontaneous model of human ovarian cancer. Gynecol Oncol 109: 115–121. 18272210 10.1016/j.ygyno.2007.12.030 PMC2387249

[7] Vermeulen K, Van Bockstaele DR, Berneman ZN (2003) The cell cycle: a review of regulation, deregulation and therapeutic targets in cancer. Cell Prolif 36: 131–149. 12814430 10.1046/j.1365-2184.2003.00266.x PMC6496723

[8] D'Andrilli G, Kumar C, Scambia G, Giordano A (2004) Cell cycle genes in ovarian cancer: steps toward earlier diagnosis and novel therapies. Clin Cancer Res 10: 8132–8141. 15623586 10.1158/1078-0432.CCR-04-0886

[9] Sun F, Fu H, Liu Q, Tie Y, Zhu J, et al (2008) Downregulation of CCND1 and CDK6 by miR-34a induces cell cycle arrest. FEBS Lett 582: 1564–1568. 18406353 10.1016/j.febslet.2008.03.057

[10] Medina R, Zaidi SK, Liu CG, Stein JL, van Wijnen AJ, et al (2008) MicroRNAs 221 and 222 bypass quiescence and compromise cell survival. Cancer Res 68: 2773–2780. 18413744 10.1158/0008-5472.CAN-07-6754 PMC3613850

[11] Ivanovska I, Ball AS, Diaz RL, Magnus JF, Kibukawa M, et al (2008) MicroRNAs in the miR-106b family regulate p21/CDKN1A and promote cell cycle progression. Mol Cell Biol 28: 2167–2174. 18212054 10.1128/MCB.01977-07 PMC2268421

[12] Siegel R, Ward E, Brawley O, Jemal A (2011) Cancer statistics, 2011: the impact of eliminating socioeconomic and racial disparities on premature cancer deaths. CA Cancer J Clin 61: 212–236. 21685461 10.3322/caac.20121

[13] Fathalla MF (1971) Incessant ovulation–a factor in ovarian neoplasia? Lancet 2: 163. 4104488 10.1016/s0140-6736(71)92335-x

[14] Smith ER, Xu XX (2008) Ovarian ageing, follicle depletion, and cancer: a hypothesis for the aetiology of epithelial ovarian cancer involving follicle depletion. Lancet Oncol 9: 1108–1111. 19012860 10.1016/S1470-2045(08)70281-X PMC2713057

[15] Roh MH, Kindelberger D, Crum CP (2009) Serous tubal intraepithelial carcinoma and the dominant ovarian mass: clues to serous tumor origin? Am J Surg Pathol 33: 376–383. 19011565 10.1097/PAS.0b013e3181868904

[16] Crum CP, McKeon FD, Xian W (2012) The oviduct and ovarian cancer: causality, clinical implications, and “targeted prevention”. Clin Obstet Gynecol 55: 24–35. 22343226 10.1097/GRF.0b013e31824b1725 PMC3319355

[17] Lee Y, Miron A, Drapkin R, Nucci MR, Medeiros F, et al (2007) A candidate precursor to serous carcinoma that originates in the distal fallopian tube. J Pathol 211: 26–35. 17117391 10.1002/path.2091

[18] Crum CP, Drapkin R, Miron A, Ince TA, Muto M, et al (2007) The distal fallopian tube: a new model for pelvic serous carcinogenesis. Curr Opin Obstet Gynecol 19: 3–9. 17218844 10.1097/GCO.0b013e328011a21f

[19] Masamha CP, Benbrook DM (2009) Cyclin D1 degradation is sufficient to induce G1 cell cycle arrest despite constitutive expression of cyclin E2 in ovarian cancer cells. Cancer Res 69: 6565–6572. 19638577 10.1158/0008-5472.CAN-09-0913

[20] Dhar KK, Branigan K, Parkes J, Howells RE, Hand P, et al (1999) Expression and subcellular localization of cyclin D1 protein in epithelial ovarian tumour cells. Br J Cancer 81: 1174–1181. 10584879 10.1038/sj.bjc.6690826 PMC2374327

[21] Courjal F, Louason G, Speiser P, Katsaros D, Zeillinger R, et al (1996) Cyclin gene amplification and overexpression in breast and ovarian cancers: evidence for the selection of cyclin D1 in breast and cyclin E in ovarian tumors. Int J Cancer 69: 247–253. 8797862 10.1002/(SICI)1097-0215(19960822)69:4<247::AID-IJC1>3.0.CO;2-X

[22] Sui L, Dong Y, Ohno M, Sugimoto K, Tai Y, et al (2001) Implication of malignancy and prognosis of p27(kip1), Cyclin E, and Cdk2 expression in epithelial ovarian tumors. Gynecol Oncol 83: 56–63. 11585414 10.1006/gyno.2001.6308

[23] Barboule N, Baldin V, S JO, Vidal S, Valette A (1998) Increased level of p21 in human ovarian tumors is associated with increased expression of cdk2, cyclin A and PCNA. Int J Cancer 76: 891–896. 9626358 10.1002/(sici)1097-0215(19980610)76:6<891::aid-ijc20>3.0.co;2-4

[24] Shimizu M, Nikaido T, Toki T, Shiozawa T, Fujii S (1999) Clear cell carcinoma has an expression pattern of cell cycle regulatory molecules that is unique among ovarian adenocarcinomas. Cancer 85: 669–677. 10091740 10.1002/(sici)1097-0142(19990201)85:3<669::aid-cncr17>3.0.co;2-f

[25] Malumbres M, Barbacid M (2005) Mammalian cyclin-dependent kinases. Trends Biochem Sci 30: 630–641. 16236519 10.1016/j.tibs.2005.09.005

[26] Stark GR, Taylor WR (2006) Control of the G2/M transition. Mol Biotechnol 32: 227–248. 16632889 10.1385/MB:32:3:227

[27] Barrette BA, Srivatsa PJ, Cliby WA, Keeney GL, Suman VJ, et al (1997) Overexpression of p34cdc2 protein kinase in epithelial ovarian carcinoma. Mayo Clin Proc 72: 925–929. 9379694 10.1016/S0025-6196(11)63362-4

[28] Marone M, Scambia G, Giannitelli C, Ferrandina G, Masciullo V, et al (1998) Analysis of cyclin E and CDK2 in ovarian cancer: gene amplification and RNA overexpression. Int J Cancer 75: 34–39. 9426687 10.1002/(sici)1097-0215(19980105)75:1<34::aid-ijc6>3.0.co;2-2

[29] Masciullo V, Scambia G, Marone M, Giannitelli C, Ferrandina G, et al (1997) Altered expression of cyclin D1 and CDK4 genes in ovarian carcinomas. Int J Cancer 74: 390–395. 9291427 10.1002/(sici)1097-0215(19970822)74:4<390::aid-ijc5>3.0.co;2-q

[30] Lim W, Jeong W, Kim J, Ka H, Bazer FW, et al (2012) Differential expression of secreted phosphoprotein 1 in response to estradiol-17beta and in ovarian tumors in chickens. Biochem Biophys Res Commun 422: 494–500. 22588173 10.1016/j.bbrc.2012.05.026

[31] Lee JY, Jeong W, Lim W, Kim J, Bazer FW, et al (2012) Chicken pleiotrophin: regulation of tissue specific expression by estrogen in the oviduct and distinct expression pattern in the ovarian carcinomas. PLoS One 7: e34215. 22496782 10.1371/journal.pone.0034215 PMC3319562

[32] Lim W, Jeong W, Kim JH, Lee JY, Kim J, et al (2011) Differential expression of alpha 2 macroglobulin in response to dietylstilbestrol and in ovarian carcinomas in chickens. Reprod Biol Endocrinol 9: 137. 21978460 10.1186/1477-7827-9-137 PMC3204285

[33] Lim W, Kim JH, Ahn SE, Jeong W, Kim J, et al (2012) Avian SERPINB11 gene: a marker for ovarian endometrioid cancer in chickens. Exp Biol Med (Maywood) 237: 150–159. 22289513 10.1258/ebm.2011.011250

[34] Ahn SE, Choi JW, Rengaraj D, Seo HW, Lim W, et al (2010) Increased expression of cysteine cathepsins in ovarian tissue from chickens with ovarian cancer. Reprod Biol Endocrinol 8: 100. 20727192 10.1186/1477-7827-8-100 PMC2931516

[35] Giles JR, Olson LM, Johnson PA (2006) Characterization of ovarian surface epithelial cells from the hen: a unique model for ovarian cancer. Exp Biol Med (Maywood) 231: 1718–1725. 17138758 10.1177/153537020623101108

[36] Giles JR, Shivaprasad HL, Johnson PA (2004) Ovarian tumor expression of an oviductal protein in the hen: a model for human serous ovarian adenocarcinoma. Gynecol Oncol 95: 530–533. 15581958 10.1016/j.ygyno.2004.07.061

[37] Garzon R, Fabbri M, Cimmino A, Calin GA, Croce CM (2006) MicroRNA expression and function in cancer. Trends Mol Med 12: 580–587. 17071139 10.1016/j.molmed.2006.10.006

[38] Bartel DP (2004) MicroRNAs: genomics, biogenesis, mechanism, and function. Cell 116: 281–297. 14744438 10.1016/s0092-8674(04)00045-5

[39] Lu J, Getz G, Miska EA, Alvarez-Saavedra E, Lamb J, et al (2005) MicroRNA expression profiles classify human cancers. Nature 435: 834–838. 15944708 10.1038/nature03702

[40] Gregory RI, Chendrimada TP, Cooch N, Shiekhattar R (2005) Human RISC couples microRNA biogenesis and posttranscriptional gene silencing. Cell 123: 631–640. 16271387 10.1016/j.cell.2005.10.022

[41] Creighton CJ, Fountain MD, Yu Z, Nagaraja AK, Zhu H, et al (2010) Molecular profiling uncovers a p53-associated role for microRNA-31 in inhibiting the proliferation of serous ovarian carcinomas and other cancers. Cancer Res 70: 1906–1915. 20179198 10.1158/0008-5472.CAN-09-3875 PMC2831102

[42] Zhang L, Volinia S, Bonome T, Calin GA, Greshock J, et al (2008) Genomic and epigenetic alterations deregulate microRNA expression in human epithelial ovarian cancer. Proc Natl Acad Sci U S A 105: 7004–7009. 18458333 10.1073/pnas.0801615105 PMC2383982

[43] Komiya T, Hosono Y, Hirashima T, Masuda N, Yasumitsu T, et al (1997) p21 expression as a predictor for favorable prognosis in squamous cell carcinoma of the lung. Clin Cancer Res 3: 1831–1835. 9815570

[44] Zirbes TK, Baldus SE, Moenig SP, Nolden S, Kunze D, et al (2000) Prognostic impact of p21/waf1/cip1 in colorectal cancer. Int J Cancer 89: 14–18. 10719725 10.1002/(sici)1097-0215(20000120)89:1<14::aid-ijc3>3.0.co;2-l

[45] Lu X, Toki T, Konishi I, Nikaido T, Fujii S (1998) Expression of p21WAF1/CIP1 in adenocarcinoma of the uterine cervix: a possible immunohistochemical marker of a favorable prognosis. Cancer 82: 2409–2417. 9635534

[46] Kapranos N, Stathopoulos GP, Manolopoulos L, Kokka E, Papadimitriou C, et al (2001) p53, p21 and p27 protein expression in head and neck cancer and their prognostic value. Anticancer Res 21: 521–528. 11299798

[47] Masciullo V, Ferrandina G, Pucci B, Fanfani F, Lovergine S, et al (2000) p27Kip1 expression is associated with clinical outcome in advanced epithelial ovarian cancer: multivariate analysis. Clin Cancer Res 6: 4816–4822. 11156240

[48] Besson A, Hwang HC, Cicero S, Donovan SL, Gurian-West M, et al (2007) Discovery of an oncogenic activity in p27Kip1 that causes stem cell expansion and a multiple tumor phenotype. Genes Dev 21: 1731–1746. 17626791 10.1101/gad.1556607 PMC1920168

[49] Song G, Bazer FW, Spencer TE (2007) Pregnancy and interferon tau regulate RSAD2 and IFIH1 expression in the ovine uterus. Reproduction 133: 285–295. 17244754 10.1530/REP-06-0092

[50] Livak KJ, Schmittgen TD (2001) Analysis of relative gene expression data using real-time quantitative PCR and the 2(-Delta Delta C(T)) Method. Methods 25: 402–408. 11846609 10.1006/meth.2001.1262

